# Health outcomes and budget impact projection of anti-PD-(L)1s in cancer care in Portugal

**DOI:** 10.3389/fpubh.2023.1133959

**Published:** 2023-05-12

**Authors:** Luís Costa, Teresa Alexandre, André Mansinho, Rita Sousa, Cláudia Vieira, Robert Hughes, Alexander Roediger, Sónia Matos Pereira, António Araújo

**Affiliations:** ^1^Department of Oncology, Hospital de Santa Maria, Centro Hospitalar Lisboa Norte, Lisbon, Portugal; ^2^Instituto de Medicina Molecular-João Lobo Antunes, Faculdade de Medicina da Universidade de Lisboa, Lisbon, Portugal; ^3^Department of Medical Oncology, Instituto Português de Oncologia de Lisboa Francisco Gentil, E. P. E, Lisbon, Portugal; ^4^Department of Medical Oncology, Instituto Português de Oncologia do Porto Francisco Gentil, Porto, Portugal; ^5^Adelphi Values PROVE, Bollington, United Kingdom; ^6^MSD International GmbH, Kriens, Switzerland; ^7^MSD Portugal, Paço de Arcos, Portugal; ^8^Department of Medical Oncology, Centro Hospitalar Universitário Santo António, Porto, Portugal

**Keywords:** anti-PD-(L)1, budget, cancer care, clinical impact, Portugal

## Abstract

**Introduction:**

PD-[L]1 inhibitors revolutionized cancer treatment but challenge the affordability of health systems. This policy-focused model aimed to estimate the health and budget impact of anti-PD-(L)1s in Portugal and inform current discussions.

**Materials and methods:**

The Health Impact Projection (HIP) model estimates clinical (life years, progression-free survival [PFS] years, and quality-adjusted life years [QALY] gained and adverse events [AEs] incurred) and economic (direct and indirect costs) outcomes in a world where cancer patients are initiating treatment with standard-of-care (SOC) versus SOC plus anti-PD-(L)1s over a 3-year time horizon. Indications included adjuvant and metastatic melanoma, non-small cell lung cancer (first and second line), metastatic triple-negative breast cancer, head and neck cancer, urothelial carcinoma, and renal cell carcinoma. Model inputs were based on publicly available literature data and expert opinion.

**Results:**

The model estimated that, over 3 years, 7,773 patients would be treated with anti-PD-(L)1s, realizing a gain of 4,787 life years, 6,901 PFS years, and 4,214 QALYs and avoiding 399 AEs. The introduction of anti-PD-(L)1s had a projected average annual impact of ≈ €108 million and a share of 20% of total cancer medicines expenditure and 0.6% of total healthcare expenditure in 2021. Although higher disease management costs are expected for patients living longer with anti-PD-(L)1s and drug acquisition costs are considerable, that is partially offset by a reduction in end-of-life costs (€611,092/year) and costs associated with patient productivity lost to cancer (€9,128,142/year).

**Discussion:**

This model highlights the significant survival and QoL benefit of anti-PD-(L)1s for cancer patients in Portugal, with a relatively low increased cost in total healthcare expenditure.

## Introduction

According to GLOBOCAN, in 2020 the incidence of cancer in Portugal in both sexes was 261.8 per 100,000 cases, and a total of 60,467 new cases and 30,168 deaths were recorded as a result of the disease (1). Cancer has been a leading cause of death in the country for the last decades, accounting for 25.5% of cases and ranking second in the leading causes of death in 2019, as reported by the Contemporary Portugal Database (Pordata) (2).

The Portuguese healthcare system comprises three subsystems: the tax-funded National Health Service (Serviço Nacional de Saúde, SNS), which provides universal coverage and a broad range of benefits for patients; the private health insurance system; and some health subsystems that provide health insurance for certain professions. Either of the three subsystems experiences constraints in drug access.

Although Portugal is committed to investing in cancer prevention and treatment and the number of patients cured or surviving with high quality of life is increasing, there is a significant delay in getting access to innovative treatments compared to other similarly developed European countries (3). Between 2017 and 2020, the median time from European Medicines Agency (EMA) approval to availability of new oncology products to patients was 753 days, compared to Germany, where this figure was 100 days (3). A delay in patient access to new drugs results in diminished patient benefits. Underlying these issues are two significant challenges for Portugal: the ageing of the population, which causes a rise in health resource requirements and chronic conditions, and healthcare cost containment policies and efficiency measures that followed the 2010–2014 economic crisis, which contributed to the delivery of less expensive treatments as a result of lower healthcare budget available compared to other European countries (4). The uneven access to innovative cancer treatments leads to potential loss of life-years. Already in 2009, a study showed that 15.3% of the disability-adjusted life years (DALYs) in Portugal were associated with cancer (5), and a more recent report from 2015 concluded that 527,651 DALYs in that year were due to oncological diseases (6). In line with the picture in Europe, the economic burden associated with cancer care in Portugal is also a concern. According to a recent study, the informal care costs related to cancer amounted to 371 million € and indirect costs to 847 million € (655 million € in productivity loss from premature mortality +192 million € in productivity loss from morbidity) in Portugal in 2018 (7). After the economic crisis, the recovery of the public health system begun, with an increasing focus on reforming the structure of the health sector toward increased access, quality, and efficiency of care.

Treatment options for cancer within the Portuguese healthcare system vary greatly depending on tumor type, stage, and location. They include chemotherapy, targeted therapy, and radiotherapy, with the first still commonly used in various cancers despite its high levels of toxicity (8). As a result, chemotherapy-treated patients often experience significant decreases in quality of life (9, 10), particularly at advanced stages of the disease, and some end up discontinuing or even refusing treatment, forgoing the usually limited survival benefits that would otherwise be gained (11, 12).

Immune checkpoint inhibitors targeting programmed death-1 and its ligand (PD-[L]1) revolutionized the therapeutic landscape in cancer over the last decade, offering improved health outcomes across a wide range of hematological and solid malignancies (13–15). Several clinical trials with these agents have shown their benefit in key indications, including a 42% decrease in the relative risk of disease progression compared to the anti-CTLA-4 ipilimumab in melanoma patients and a 51% lower probability of death compared to placebo in non-small cell lung cancer (NSCLC) patients (16, 17). The tolerability profile of anti-PD-(L)1 monotherapies appears to be more favorable than standard of care (SOC) treatments such as chemotherapy, with significantly lower risk of all-and high-grade fatigue, sensory neuropathy, diarrhea, and hematologic toxicity, lower risk of all-grade anorexia, nausea, and constipation, and also lower risk of treatment discontinuation (18).

Despite the acknowledged value of this new immune-oncology therapeutic class, the rapidly growing number of patients eligible for these therapies seems to challenge the long-term affordability of health systems given their potential for use in several cancer types (19–21).

The Health Impact Projection (HIP) model is a policy-focused, macro-oriented model that assesses the budget impact of the introduction of PD-(L)1 inhibitors, while also incorporating a wider value assessment encompassing the health benefits achieved by patients undergoing treatment (22). The model compares key clinical (life years, progression-free survival [PFS] years, patient quality-adjusted life years [QALY] gained and adverse events [AEs] incurred) and economic (direct and indirect healthcare costs) outcomes in a world where cancer patients are treated only with SOC (reference scenario) versus with a mix of SOC and anti-PD-(L)1 treatments (new scenario). The HIP model has been previously adapted for other country-specific circumstances, where the clinical and economic impact of anti-PD-(L)1s was explored, namely Italy, Austria, Belgium, Slovenia, and more recently Ireland (23–27). They have also shown the value of anti PD-(L)1s in cancer care and in the expenditure of healthcare systems often struggling with uncertainty in budget allocation and pressure to reduce costs.

The aim of this study was to estimate the potential health and budget impact of the anti-PD-(L)1 class in Portugal to inform current discussions among stakeholders and policymakers, contributing to decision-making and budget planning.

## Materials and methods

The HIP model used partitioned survival modeling to estimate key survival outcomes and budget impact (28). Partitioned survival modeling was preferred to Markov modeling for consistency with the literature and due to its less stringent data requirements and programming simplicity (28). Survival outcomes attained with anti–PD-1/PD-L1 treatments were modeled for the entire class, rather than for each product individually. Due to the lack of available data for some products within indications, the model assumed that the survival outcomes associated with the anti–PD-1/PD-L1 products, for which data were available, were representative of the whole anti–PD-1/PD-L1 class (although, in reality, health benefits may vary considerably from molecule to molecule). In indications where data on multiple anti–PD-1/PD-L1 products were available, a conservative approach was taken whereby only data on the product which achieved the lowest gain in median time to progression, versus the comparator in a trial, was used as an estimate for the entire class in that indication. Consequently, the health impact of the anti–PD-1/PD-L1 class is likely to be underestimated.

The study considered patients entering the model over a three-year time frame (2021–2023) and captured 5 years of costs and health outcomes for each patient cohort (2024+).

Six cancer types were selected based on the current and expected availability of anti-PD-(L)1s as treatment options for those indications within the study time period: adjuvant and metastatic melanoma, NSCLC (first and second line), metastatic triple-negative breast cancer, head and neck cancer, urothelial carcinoma, and renal cell carcinoma.

Model inputs were based on publicly available data, literature data, and expert opinion as follows:

- Clinical inputs of anti-PD-(L)1 and SOC treatments, including PFS, overall survival, health-related quality of life (HRQoL), and adverse events in each indication were retrieved from clinical trial data of anti-PD-(L)1s in each specific indication.
- The treatment options included and used within each indication are aligned with the availability under EMA labelling, expected reimbursement decisions made in Portugal, and ongoing clinical trials.
- Total costs of the anti PD-(L)1 class were estimated using costs for each individual treatment within each class and combining these to estimate an average total cost, weighted by the market share of each treatment.
- Direct and indirect costs were included in the model. Direct costs included the costs of administering the drugs, drug procurement, disease management, PD-L1 biomarker testing (when applicable), palliative care, and AEs associated with treatment, and were chosen to ensure consistency with budget impact analysis good practice (22). Indirect costs included the costs to society due to loss of productivity (estimated as hours of work lost), transport, and complementary treatments as a result of illness from cancer.
- Epidemiological data of each cancer type was used to estimate the number of patients eligible to receive anti-PD-(L)1 treatments in each indication.
- Market share data and data on cancer incidence in Portugal in each indication were combined to estimate the proportion of patients undergoing treatment with anti-PD-(L)1s rather than SOC. The HIP model considered only patients newly diagnosed in advanced stages of disease, as this is the indication for anti-PD-(L)1 eligibility. These data were also used to estimate the proportion of patients undergoing treatment with each individual SOC and anti-PD-(L)1. Given the lack of published sources on anti-PD-(L)1 market shares in Portugal, the HIP model relied on assumptions based on local market research studies.
- Resource use inputs were used to estimate the healthcare resources required to treat each patient with each treatment considered in the model and included the longest treatment duration (retrieved from the European Medicines Agency [EMA] product labels and clinical trials.). The longest treatment duration, instead of the median, was used as a cap on the absolute maximum length of patient treatment, with patients assumed to cease treatment upon disease progression prior to this cap.
- AEs incurred with anti-PD-(L)1s and comparators in each indication were retrieved from the respective clinical trials.

## Results

### Health impact

The Portuguese adaptation of the HIP model found that the introduction of PD-(L)1s annually over a three-year period across eight indications carried improvements for the entire patient population in all the modeled health outcomes.

The model estimated that in the considered time period, 12,890 patients would be eligible for anti-PD-(L)1 treatment in the country. Of these, an estimated 7,773 patients would be treated with these agents, obtaining clinical gains including an additional 4,787 life years (+23% of relative gains), 6,901 PFS years (+98% of relative gains), and 4,214 QALYs (+30% of relative gains) and avoiding 399 AEs (+4% of relative gains) for patients who initiated treatment over 3 years versus a world where cancer patients are treated with SOC (Table 1). This shows that, on average, anti-PD-(L)1s provide over 7 months of additional life per patient across all indications and improve quality of life (QoL), as patients live longer in a progression-free disease state. These improvements come with the additional benefit of more tolerable treatments due to the reduction of AEs.

**Table 1:** Total health outcomes of anti-PD-(L)1 in Portugal in 2021–2023.

Absolute change	Gains with anti-PD-(L)1	Relative gains
4,787	Life years gained	+23%
4,214	QALYs gained	+30%
6,901	PFS life years gained	+98%
399	AEs avoided	+4%

AE, adverse event; PFS, progression-free survival; PD-1, programmed cell death protein-1; PD-L1, programmed cell death-ligand 1; QALY, quality-adjusted life year.

The estimated number of patients treated was based on Globocan data sets and internal estimations of eligible patients and expected uptake. While rounding could be applied to show that this is an estimation based on a series of assumptions, the transparency of the exact values used in the model was assumed to be of greater value.

### Economic impact

Although the health impact of the anti-PD-(L)1 class is undoubtedly relevant, this cannot be viewed without the financial impact this innovation brings. To assess the overall value of a new class of therapies, its health gains need to be analyzed alongside the budget impact of a new class of therapies. In the present model, the public and budget impact of the anti-PD-(L)1 class was driven by the indication-specific population sizes.

The HIP predicted that, the average cost of bringing anti-PD-1/PD-L1 to market across the eight indications in the five-year period would amount to around €108 million per year. The economic impact was expected to grow over time, from around €90 million in 2021 to around €124 million in 2023, an increase that is related to the increasing number of patients treated with anti-PD-(L)1s (Table 2).

**Table 2:** Yearly breakdown of the healthcare economic impact of the anti-PD-(L)1 class.

	2021	2022	2023	2024+	Average
Economic impact (€)	89,930,968	110,970,853	124,435,262	80,110,138	108,445,694

PD-1, programmed cell death protein-1; PD-L1, programmed cell death-ligand 1.

Breakdown of the economic impact of the anti PD-(L)1 class by cost category showed that drug acquisition costs represented the largest component of the total economic impact of the class expected over the considered time horizon (average of €111,841,159 per year), corresponding on average to 103% of its total economic impact (Table 3). However, this is partly offset by expected reductions in end-of-life costs over the 3 years, by an average of €611,092 a year. Disease management costs showed a steady rise over time because patients treated with anti-PD-(L)1s have longer survival and are treated for longer. Therefore, patients receiving anti-PD-(L)1s incur greater disease management costs later, resulting in an increase in costs.

**Table 3:** Breakdown of the economic impact of the anti PD-(L)1 class by cost category.

	Yearly economic impact across all indications (€)				
Cost category	2021	2022	2023	2024 +	Average
Disease management costs	2,387,561	4,415,301	6,951,575	20,243,568	4,584,812
Administration costs	708,542	1,091,192	1,298,746	1,245,380	1,032,827
Drug acquisition costs	92,272,420	114,361,763	128,889,294	92,763,252	111,841,159
PD-(L)1 testing costs	155,485	151,283	154,604	0	153,791
Indirect costs	−5,552,313	−9,011,403	−12,820,709	−34,179,742	−9,128,142
AE costs	474,167	595,382	647,468	554,933	572,339
End-of-life costs	−514,894	−632,666	−685,716	−517,253	−611,092
Total	89,930,968	110,970,853	124,435,262	80,110,138	108,445,694

AE, adverse event; PD-1, programmed cell death protein-1; PD-L1, programmed cell death-ligand 1.

Although results showed a 4% decrease in the number of AEs experienced by patients, the costs related to these events increased over time for the same reason as disease management costs. As patients are alive for longer with the introduction of anti-PD-(L)1s, they incur more AEs, and the cost of these AEs is considered as long as active treatment is administered. However, the superior toxicity profile associated with anti-PD-(L)1s is expected to result in improved QoL for patients.

Table 3 also shows a reduction in indirect costs with the introduction of anti-PD-(L)1s in Portugal, corresponding to a reduction in patient productivity lost to cancer associated with anti-PD-(L)1s. The monetary value of increased time at work is estimated at an average of €9.1 million every year. Additionally, it can be noted that PD-(L)1 testing costs are zero for the year 2024+. This is because no new patients are entering the model in 2024 + .

Overall, although higher disease management and administration costs are expected due to patients living longer with anti-PD-(L)1s and drug acquisition costs are considerable, some of that is partially offset by a reduction in end-of-life costs and costs of patient productivity lost to cancer.

**Figure 1:**
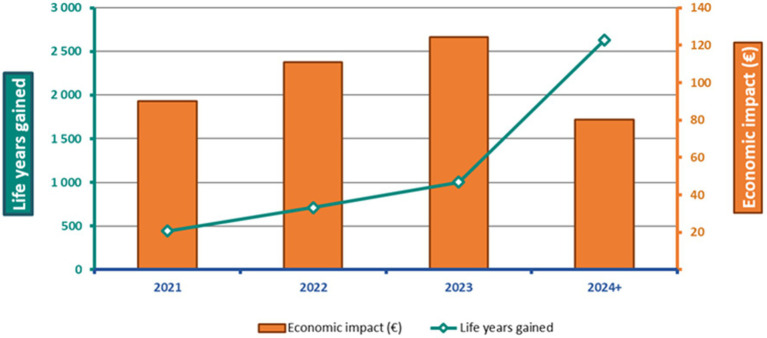
Health and economic impact of anti-PD-(L)1s over 4 years.

Figure 1 schematizes the health and economic impact of the anti-PD-(L)1 class over 4+ years, highlighting that the majority of healthcare costs for patients are incurred within the first three years modeled, while health outcomes continue to be incurred over a longer time frame.

**Figure 2:**
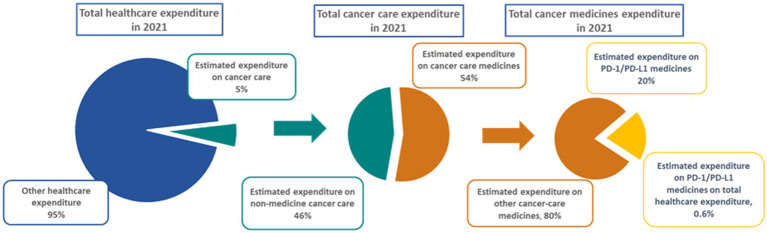
Estimated expenditure of anti-PD-(L)1s in Portugal in 2021. PD-1, programmed cell death protein-1; PD-L1, programmed cell death-ligand 1.

Figure 2 shows the investment required by Portugal to bring the anti-PD-(L)1 class to the market in 2021, with a detailed breakdown of the estimated expenditure of the class as a proportion of the predicted total healthcare budget and total medicines expenditure. The HIP estimated that an investment equivalent to 0.6% of the total healthcare expenditure was required in 2021 to help fund the use of anti-PD-(L)1s, corresponding to a projected share of 20% of cancer medicines expenditure.

## Discussion

To the authors’ knowledge, this study represents the first systematic effort to estimate the budget impact and health outcomes of the anti-PD-(L)1 class in Portugal. As such, it provides a unique and comprehensive framework to predict the outcomes associated with this novel class of immuno-oncology products, providing data to help inform health policy decisions about cancer care. The model was not intended to provide detailed information about specific treatments or to compare across individual treatments.

The model focused on a three-year time horizon (2021–2023) and included eight indications in six cancer types. During this time frame, the use of anti-PD-(L)1s is projected to be still growing and was not widespread in some tumors, due to reimbursement constraints (e.g., breast cancer).

The model has allowed for each cohort of patients to incur benefits for up to 5 years following treatment initiation. For projections beyond this length of time, a considerable additional level of uncertainty in the persistence of health gains was foreseen, and therefore it was decided that a 5-year period would be sufficient to reflect the longer-term benefits while remaining mindful of the potential uncertainty resulting from data extrapolation.

The HIP adaptation for Portugal estimates that anti-PD-(L)1 treatments are expected to provide improvements in health outcomes, with gains gradually increasing between 2021─2023. Similar results have been reported in Austria, Belgium, Italy, Slovenia, and more recently Ireland, clearly highlighting the added value that this new class of innovative treatments represents for cancer patients (23–27).

Financially, the HIP estimated that in 2021 the budget impact of the class in Portugal was expected to represent a somewhat relevant portion of the total expenditure on cancer medicines (20%), but a small portion of the total healthcare expenditure (0.6%). Although direct costs of introducing these treatments in the Portuguese market are expected to rise over the next years in parallel with the increased uptake of PD-(L)1 inhibitors, growing ageing and longevity of the population, and higher incidence of cancer, this will be partly offset by a reduction in the burden of end-of-life costs and indirect costs related to patient productivity lost to cancer.

It should be noted that, although the study considers 3 years’ worth of patients entering the model, it included 5 years’ worth of costs and health outcomes for each patient cohort, captured in the 2024+ analysis. By projecting budget impact over a 4+ year period, the HIP model can help inform budget planning for innovative cancer medicines, highly relevant in a country where the healthcare system struggles with resource and budget allocation and is under great pressure to increase health system efficiency and contain costs. According to the OECD/European Observatory on Health Systems and Policies report of 2021, the largest share of healthcare spending in Portugal in 2019 was on outpatient care (46%; €1,074 *per capita*, only slightly above the EU average of €1,022 *per capita*) (29), clearly surpassing the expenditure on inpatient care (26%; €598) and pharmaceutical care (19%; €443; both considerably below the EU averages of €1,010 and €630, respectively) (4). The same source highlighted that, between 2010 and 2019, government spending on health decreased by around 5.6% (from 66.6 to 61.0%), being almost 20% below the EU average (79.7%) (4). Although spending has recovered since the economic crisis, in 2019 Portugal spent €2,314 *per capita* on healthcare (9.5% of the gross domestic product), about one third less than the EU average (€3,521) (29). Given the need to care for an ageing population with rising health needs and chronic conditions as one of the most significant challenges faced by the health system regarding financial sustainability, the Portuguese adaptation of the HIP model can provide valuable insights to stakeholders and policymakers on the economic impact of anti-PD-(L)1 treatments on cancer care costs and on the country’s healthcare system budget, contributing to decision-making and budget planning.

### Constraints and limitations of the HIP model

The HIP model was developed with a wide scope to ensure maximum utility for policymakers. Given the uncertainty that surrounds future trends in immuno-oncology, the model provides a basis for independent and collaborative discussions with stakeholders.

The HIP relies on a set of assumptions whose validity is paramount to ensuring that the study’s results are also valid. For this reason, a wide range of experts were involved in assessing the appropriateness of structural and data assumptions made. Several of the assumptions were made because of constraints in the data or resources available. A crucial assumption is that the budget impact of the anti-PD-(L)1 class is based on publicly available list prices, as informed by the respective national formularies. Due to this, results are likely to be an overestimation of actual costs incurred by payers, as this conservative approach does not accurately reflect the effect of flexible access agreements between companies and payers or governments, which often involve confidential rebates that bring down the effective acquisition price of medicines.

The method used to model survival in the HIP relies on visual inspection as key criterion to select the hazard ratios and time period cut-off point along each survival curve to adopt in the model. This method is simple and intuitive, although it lacks the rigor of more advanced statistical techniques. Visual inspection was chosen as primary technique because of the broad objectives of this study: as one product’s survival curve is taken as representative of the whole anti-PD-(L)1 class in each indication, performing advanced statistical techniques to ensure great accuracy was not necessary. The purpose of extrapolation was never to accurately reflect the survival experience associated with a single product, but rather to gain some insight as to what survival with the average anti-PD-(L)1 product in that indication might look like.

HIP is not a cost-effectiveness analysis nor should be used to infer the cost-effectiveness of the anti-PD-(L)1 class. This is due to a number of reasons, starting with its short time horizon, which is not adequate for providing a realistic estimate of the long-term health outcomes of anti-PD-(L)1s. Secondly, the comparators used in the HIP are not necessarily the most relevant alternatives to anti-PD-(L)1s, since in many cases the SOCs specified for an indication are simply the most widely used products and not the next best alternative. Thirdly, simplified assumptions were made in the HIP regarding future health gains and how they are modeled compared to standard cost-effectiveness analyses. These methods were chosen due to their simplicity and fit for purpose given the high-level objectives of the model, rather than focusing on precision and estimation of uncertainty. Lastly, due to its structure more closely aligned to that of a budget impact model (BIM), the HIP does not calculate long-term health benefits with sufficient detail and stringency to perform a cost-effectiveness analysis. For all the above-mentioned reasons, HIP should not be used to infer incremental cost-effectiveness ratios from its results. Instead, it can be used to estimate the budget impact and patient use of the anti-PD-(L)1 class as a tool to inform discussions with policymakers, whilst also providing a wide-ranging background of the health benefits of the class within a short time horizon.

Finally, the economic results from the 2024+ time horizon should not be directly compared to other years in the model, as this is not the cost of one additional year, but the costs of all cohorts for a full 3 years after treatment start, beyond the three-year time horizon in the model. To this end, the average annual costs throughout the cost results do not include the 2024+ year costs. However, for completeness the total costs do include the 2024+ year.

## Conclusion

This model highlights the significant survival and QoL benefits of anti-PD-(L)1s for cancer patients in Portugal, with a manageable low increased cost in the total healthcare expenditure.

## Data availability statement

The original contributions presented in the study are included in the article/supplementary material, further inquiries can be directed to the corresponding author.

## Author contributions

RH carried out the analysis. LC, TA, AM, RS, CV, AR, SP, and AA discussed the results and critically reviewed the manuscript. All the authors equally contributed to the manuscript and approved the final version.

## Funding

This study was supported by MSD Portugal.

## Conflict of interest

LC participated in advisory boards sponsored by MSD. AM received honoraria as consultant/speaker from Amgen, Astellas, Bayer, B. Braun, Bristol Myers-Squibb, Janssen, Merck-Serono, Merck Sharp & Dohme, Novartis, OM Pharma, Pfizer, Pierre Fabre, Roche, and Servier; travel/logistics support from Amgen, Astellas, Bayer, Bristol Myers-Squibb, Janssen, Merck-Serono, Merck Sharp & Dohme, Novartis, OM Pharma, Pfizer, Pierre Fabre, Roche, and Servier; and research funding from Bayer.

RS received honoraria as consultant/speaker from Roche, Merck Sharp & Dohme, Novartis, Pierre-Fabre, Bristol-Myers Squibb, Astrazeneca, Glaxosmith, Lilly, Pfizer, and Tesaro.

CV performed consulting or advisory activities for MSD, Bristol-Myers Squibb, Merck Serono, Novartis, F. Hoffmann-La Roche Ltd., Lilly and Grünenthal and received reimbursement for travel expenses from F. Hoffmann-La Roche Ltd., MSD, BMS, Pfizer, and Novartis.

RH is an employee of Adelphi Values Ltd. Adelphi Values Ltd. received reimbursement from MSD for the conduct of this study.

AR and SP are employees of MSD and may own stock and/or hold stock options in Merck & Co., Inc., Kenilworth, NJ, USA.

AA performed consulting/advisory activities for Merck Sharp & Dohme, Bristol Myers Squibb, AstraZeneca, Janssen, Pfizer, IPSEN, Takeda, Boehringer Ingelheim; research funding for Astellas, Janssen; and expert testimony for Merck Sharp & Dohme, Bristol Myers Squibb, AstraZeneca, Janssen, Pfizer, IPSEN, Takeda, and Boehringer Ingelheim.

The authors declare that this study received funding from MSD Portugal and MSD International Business GmbH. The funder had the following involvement in the study: funding in model development, contributing with local data and expertise, and commenting on the paper. The remaining author declares that the research was conducted in the absence of any commercial or financial relationships that could be construed as a potential conflict of interest.

## Publisher’s note

All claims expressed in this article are solely those of the authors and do not necessarily represent those of their affiliated organizations, or those of the publisher, the editors and the reviewers. Any product that may be evaluated in this article, or claim that may be made by its manufacturer, is not guaranteed or endorsed by the publisher.
